# The impact of constipation management on the quality of life of Alzheimer’s patients

**DOI:** 10.1002/alz.083777

**Published:** 2025-01-09

**Authors:** Viacheslav Viktorovich Sushko, Viktor Vasilievich Sushko

**Affiliations:** ^1^ National University “Odessa Law Academy”, Odessa Ukraine; ^2^ Odessa National Maritime University, Odessa Ukraine

## Abstract

**Background:**

Patients with Alzheimer’s disease due to the peculiarities of this disease do not report constipation.

**Method:**

In a study of 11 women with Alzheimer’s disease, aged 70‐75 years, we found that all of them had a tendency towards constipation. We divided the patients into two groups: a control group of 6 women who received help when they complained of constipation symptoms and an experimental group of 5 female patients who were routinely assessed by caregivers on their fecal status using the Bristol Scale. Through a combination of diet, physical exercises, and medication, we were able to maintain the type of feces in the experimental group at a level of 3‐5 on the Bristol Scale. The Neuropsychiatric Inventory Questionnaire (NPI‐Q) was filled out by nursing staff under medical supervision every month, and both groups of patients completed a monthly Quality of Life Scale.

**Result:**

After 3 months, we found that the patients in the control group did not seek help in a timely manner, despite receiving instructions. In contrast, the experimental group showed improvements in sleep, mood, and social communication. The NPI‐Q results showed that sleep normalized in the experimental group within 3‐4 weeks, with no early awakenings, difficulty falling asleep, or daytime sleep observed until the end of the study. Additionally, after 4‐5 weeks, the mood of these patients improved, and they showed less anxiety. They also became more cooperative and willing to help with their own care. In contrast, the control group experienced periodic anxiety, bad mood, dysphoria, sleep disorders, and resistance to nursing care. Furthermore, the experimental group subjectively assessed their quality of life higher than the control group.

**Conclusion:**

In conclusion, patients with Alzheimer’s disease require additional methods of control for the prevention and early detection of constipation. The timely application of these methods can greatly improve their subjective assessment of their quality of life, as well as their sleep, mood, and social communication. One such method is the use of the Bristol Scale by medical staff.

